# Chronic administration of P2X7 receptor antagonist JNJ-47965567 delays disease onset and progression, and improves motor performance in ALS SOD1^G93A^ female mice

**DOI:** 10.1242/dmm.045732

**Published:** 2020-10-30

**Authors:** Cristina Ruiz-Ruiz, Nuria García-Magro, Pilar Negredo, Carlos Avendaño, Anindya Bhattacharya, Marc Ceusters, Antonio G. García

**Affiliations:** 1 Instituto Teófilo Hernando, Facultad de Medicina, Universidad Autónoma de Madrid, Madrid 28029, Spain; 2 Departamento de Farmacología, Facultad de Medicina, Universidad Autónoma de Madrid, Madrid 28029, Spain; 3 Departamento de Anatomía, Histología y Neurociencia, Facultad de Medicina, Universidad Autónoma de Madrid, Madrid 28029, Spain; 4 Instituto de Investigación Sanitaria, Hospital Universitario de La Princesa, Madrid 28006, Spain; 5 Neuroscience Therapeutic Area, Janssen Research and Development LLC., 3210 Merryfield Row, San Diego, CA 92121, USA; 6 Neuroscience Therapeutic Area, Janssen Research and Development, a Division of Janssen Pharmaceutica NV, Beerse B-2340, Belgium

**Keywords:** Amyotrophic lateral sclerosis, SOD1^G93A^ mice, Neuroinflammation, P2X7 receptor, Purinergic signaling

## Abstract

Neuroinflammation is one of the main physiopathological mechanisms of amyotrophic lateral sclerosis (ALS), produced by the chronic activation of microglia in the CNS. This process is triggered by the persistent activation of the ATP-gated P2X7 receptor (P2RX7, hereafter referred to as P2X7R). The present study aimed to evaluate the effects of the chronic treatment with the P2X7R antagonist JNJ-47965567 in the development and progression of ALS in the SOD1^G93A^ murine model. SOD1^G93A^ mice were intraperitoneally (i.p.) injected with either 30 mg/kg of JNJ-47965567 or vehicle 4 times per week, from pre-onset age (here, postnatal day 60; P60) until study endpoint. Body weight, motor coordination, phenotypic score, disease onset and survival were measured throughout the study, and compared between vehicle- and drug-injected groups. Treatment with the P2X7R antagonist JNJ-47965567 delayed disease onset, reduced body weight loss and improved motor coordination and phenotypic score in female SOD1^G93A^ mice, although it did not increase lifespan. Interestingly, neither beneficial nor detrimental effects were observed in males in any of the analyzed parameters. Treatment did not affect motor neuron survival or ChAT, Iba-1 and P2X7R protein expression in endpoint individuals of mixed sexes. Overall, chronic administration of JNJ-47965567 for 4 times per week to SOD1^G93A^ mice from pre-onset stage altered disease progression in female individuals while it did not have any effect in males. Our results suggest a partial, yet important, effect of P2X7R in the development and progression of ALS.

## Abstract (precis)

**Summary:** This study implicates the P2X7 receptor (P2X7R) in amyotrophic lateral sclerosis progression in mice and shows that the effectiveness of treatment with P2X7R antagonists is sex dependent.

## INTRODUCTION

Amyotrophic lateral sclerosis (ALS) is an adult-onset and heterogeneous neurodegenerative disorder characterized by gradual loss of muscle function, spasticity and weakness, cramps and muscle wasting, speech and swallowing impairment, compromised respiration, progressive paralysis and death within 3-5 years after diagnosis (Calvo et al., 2014; Hardiman et al., 2017). So far, riluzole and edaravone are the only two approved drugs that show very modest clinical benefits in patients (Doble, 1996; Cruz, 2018). Therefore, this fatal disease still remains incurable, and there is urgent need to discover new drugs that can improve the quality of life and life expectancy of patients suffering from this devastating disease.

Multiple signaling pathways converge to cause the progressive degeneration of upper motor neurons (neurons projecting from the motor cortex to the brainstem and spinal cord) and lower motor neurons (neurons projecting from the brainstem or spinal cord to muscle), the cause for the phenotypic symptoms in ALS patients (Bonafede and Mariotti, 2017; Hardiman et al., 2017). One of the involved pathways is chronic neuroinflammation, which has been found in patients with ALS, post-mortem samples and rodent models of ALS (Corcia et al., 2012; Brites and Vaz, 2014).

The purinergic P2X7 receptor (P2RX7, hereafter referred to as P2X7R) plays a key role in the neuroinflammatory process. The activation of the receptor through high concentrations of ATP released by damaged neurons results in the assembly of the NLRP3 inflammasome complex in microglia and the subsequent release of pro-inflammatory cytokines, such as IL-1β (DiSabato et al., 2016; Swanson et al., 2019). The upregulation of P2X7R in ALS patients suggests its implication in disease progression (Yiangou et al., 2006). Moreover, stimulation of P2X7R with ATP or 3′-O-(4-Benzoyl)benzoyl ATP (BzATP) triggers a neurotoxic phenotype in astrocytes, inducing motor neuron death which is prevented by the P2X7R antagonist Brilliant Blue G (BBG) (Gandelman et al., 2010). These *in vitro* experiments also point out to a role of the P2X7R receptor in ALS disease. Furthermore, ATP stimulation of the microglial P2X7R from SOD1^G93A^ mice promotes the stimulation of NADPH oxidase 2 (NOX2) and kinases ERK1/2, with a concomitant increase of ROS (Apolloni et al., 2013b). However, short stimulation of P2X7R causes the activation of autophagy and upregulation of anti-inflammatory markers in microglia of SOD1^G93A^ mice (M2 microglia), whereas persistent stimulation impairs the autophagic flux that might correspond to the change to a pro-inflammatory phenotype (M1 microglia), suggesting a dual functional role of the receptor in the pathway (Fabbrizio et al., 2017). Thus, P2X7R seems to play a major role in ALS pathogenesis. In this context, receptor blockade with antagonists has been proposed as a new therapeutic approach to treat the disease.

**Fig. 1.**
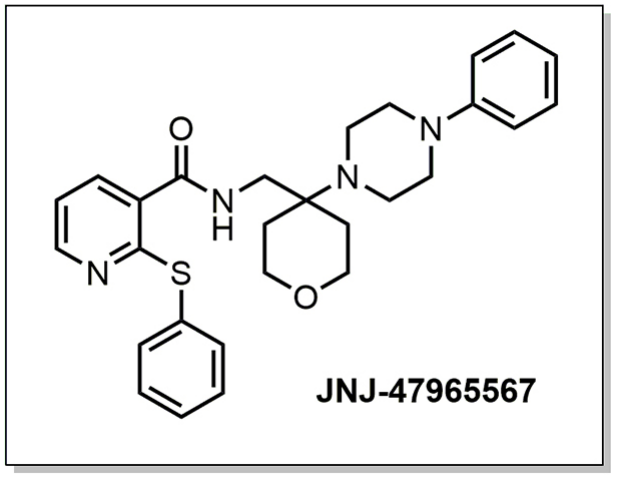
**Molecular structure of compound JNJ-47965567.** The compound was administered at a concentration of 30 mg/kg in 30% SBE-β-cyclodextrin.

This hypothesis has already been tested *in vivo* in the SOD1^G93A^ mouse model of ALS (summarized in Table 1), the most used model in preclinical stages owing to its high resemblance to the human pathology (Gurney et al., 1994; Philips and Rothstein, 2015; Lutz, 2018). In a first study, Cervetto and colleagues (Cervetto et al., 2013) showed that treatment with BBG (i.p., 45.5 mg/kg, every 2 days) starting at postnatal day 90 (P90) improved motor coordination in males and females and reduced body weight loss in males. In a second study, BBG was administered at the higher dose of 250 mg/kg, 3 times per week. When treatment was initiated at P100, delayed disease onset, improved motor performance, reduced microgliosis and enhanced motor neuron survival were observed. However, this was not the case when treatment was initiated at earlier stages (Apolloni et al., 2014). A lower BBG dose (45.5 mg/kg, 3 times per week) was used in a third study, starting at disease pre-onset time, i.e. P62-P64. Reduction in body weight loss and survival was observed in females but not in males, while motor performance was unaffected in either sex (Bartlett et al., 2017). Altogether, these studies suggest that P2X7R plays an important role in ALS progression. However, due to the low selectivity of BBG (Bo et al., 2003; Seyffert et al., 2004; Qiu and Dahl, 2009; Jo and Bean, 2011), the efficacy of other antagonists was then evaluated. Application of the more-potent and selective P2X7R blocker A-804598 from P100 onwards (30 mg/kg, 5 times per week), did not produce any differences in motor performance, disease onset or survival (Fabbrizio et al., 2017). Similar results were obtained after administration of the P2X7R antagonist JNJ-47965567 (Fig. 1; 30 mg/kg, 3 times per week) from P100 onwards (Ly et al., 2020).

**Table 1.**
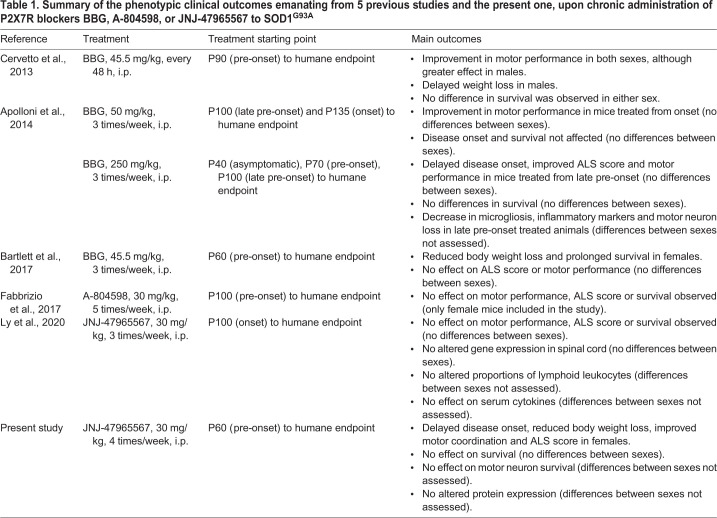
**Summary of the phenotypic clinical outcomes emanating from 5 previous studies and the present one, upon chronic administration of P2X7R blockers BBG, A-804598, or JNJ-47965567 to SOD1^G93A^**

Despite the negative results obtained by Ly and collaborators (Ly et al., 2020), we decided to further evaluate the effects of this brain-penetrant antagonist in SOD1^G93A^ mice because JNJ-47965567 had shown promising effects in models of mania and neuropathic pain, as well as high potency, selectivity and capacity to block IL-1β release (Bhattacharya et al., 2013). The aim of this present study was to evaluate the effects of chronic administration of 30 mg/kg of JNJ-47965567, 4 times per week from postnatal day 60 (P60) or pre-onset. This treatment was able to delay disease onset, reduce body weight loss, improve motor coordination and neurological scores in female SOD1^G93A^ mice, although no effects were observed in male mice.

## RESULTS

### Treatment with JNJ-47965567 delays disease onset in female SOD1^G93A^ mice

**Fig. 2.**
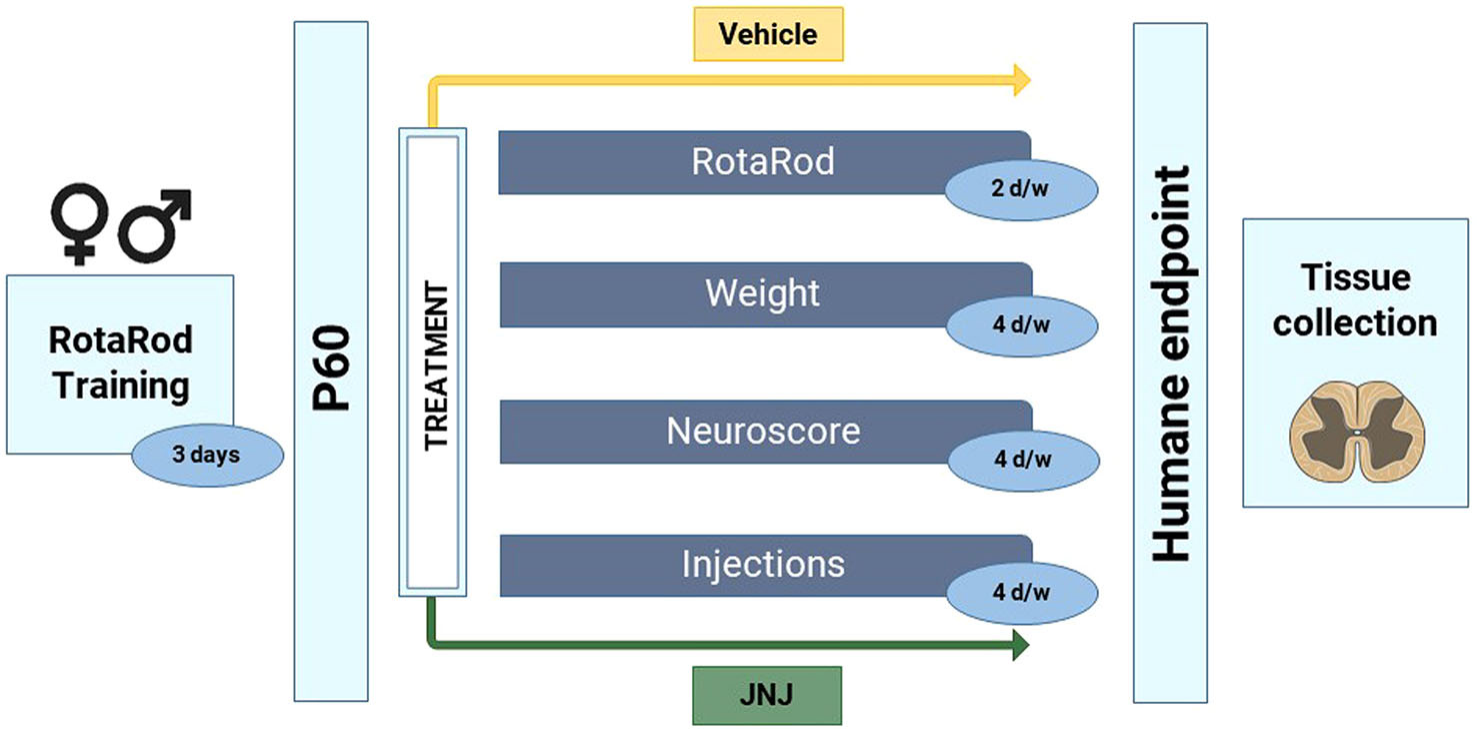
**Protocol followed for the blinded chronic i.p. administration of vehicle (30% SBE-β-cyclodextrin) and JNJ-47965567.** Animals were trained for 3 consecutive days in the RotaRod apparatus just before the commencement of the treatment. At P60, mice were divided into two treatment groups (Vehicle or JNJ-47965567) and administrations continued 4 times per week until the humane endpoint. Disease progression was followed according to the protocol by assessing motor coordination with RotaRod, by measuring body weight and according to the phenotypic NeuroScore test (see Materials and Methods). At the humane endpoint, mice were euthanized, and spinal cords were dissected and stored until used. All tests were blinded for the investigators involved.

**Fig. 3.**
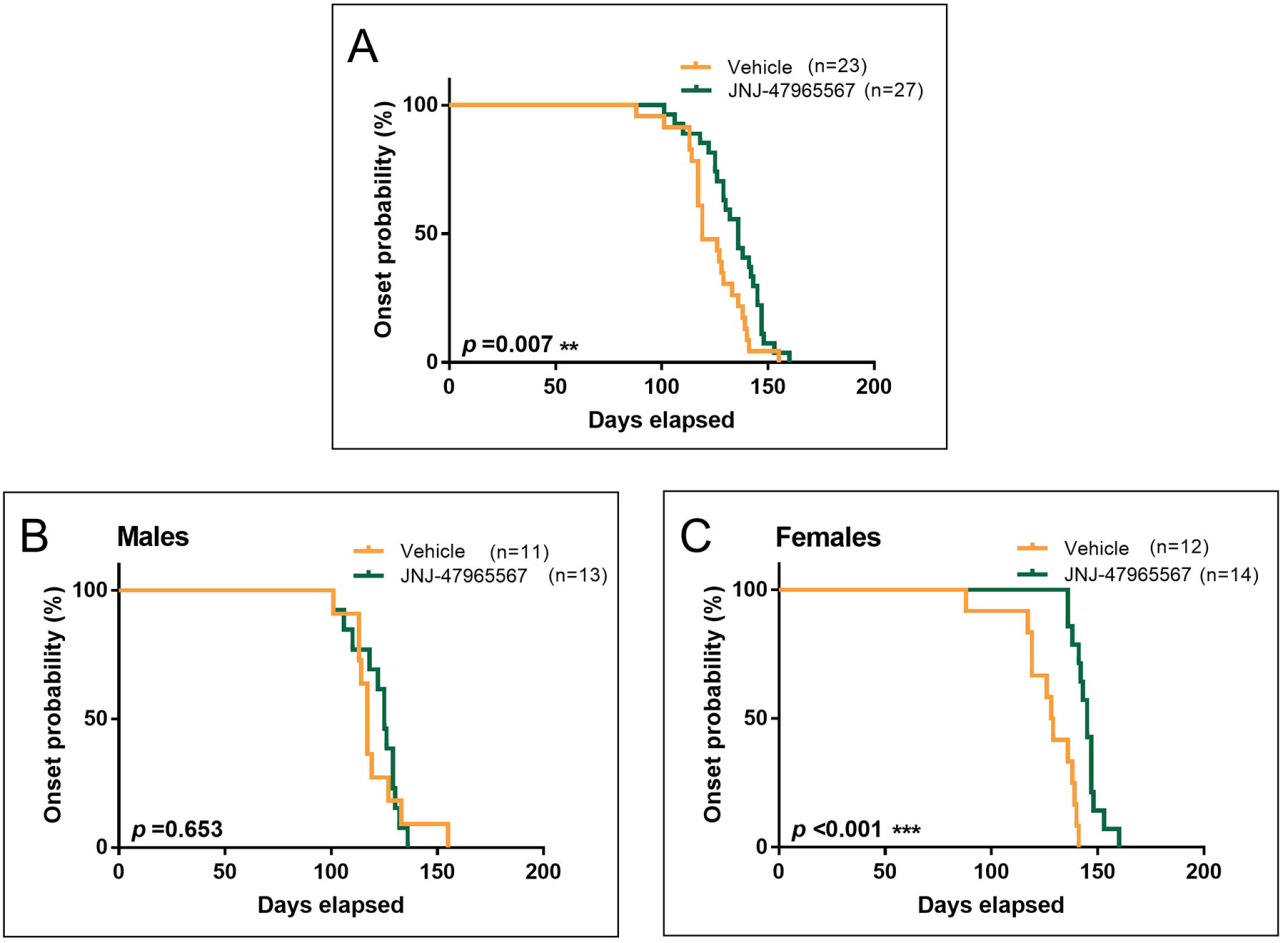
**Disease onset of SOD1^G93A^ ALS mice administered i.p. 4 times per week with vehicle (orange) or 30 mg/kg JNJ-47965567 (green).** Disease onset was defined as the time mice were losing weight for 3 consecutive days. (A) Kaplan–Meier plot showing disease onset probability in the whole group of mice. (B) Kaplan–Meier plot showing onset probability in the subset of male mice. (C) Kaplan–Meier plot showing onset probability in the subset of female mice. Data analysis was performed using Cox regression, introducing age at first injection as co-variables. Differences were considered as statistically significant at *P*<0.05.

To assess whether JNJ-47965567 treatment can delay disease onset in SOD1^G93A^ mice, 24 males and 26 females were i.p. injected from pre-symptomatic stage (P60) until the humane endpoint was reached. Of these, 11 males and 12 females were treated with vehicle (30% SBE-β-cyclodextrin), and 13 males and 14 females with 30 mg/kg of JNJ-47965567, administered i.p. 4 times per week (Fig. 2). Disease onset was defined as the day of consecutive weight loss after three weight measurements without recovery, a direct sign of muscle mass loss, degeneration and disease progression (Ludolph et al., 2010). Kaplan–Meier plots of disease onset indicates clear differences upon statistical comparisons (Fig. 3). Treatment with JNJ-47965567 delayed disease onset in SOD1^G93A^ mice when both sexes were analyzed together (Fig. 3A; 123.7±3.0 days for vehicle and 134.0±2.7 for treated mice, *P*=0.007). However, when such analysis was performed separately, treatment did not modify disease onset in the male subset of the animals (Fig. 3B; 120.5±4.2 for vehicle and 122.2±2.9 for treated mice, *P*=0.653). Strikingly, treatment significantly delayed disease onset in females, i.e. the difference observed was greater than when both sexes were analyzed as a whole (Fig. 3C; 126.7±4.3 days for vehicle and 144.9±1.7 for treated mice, *P*<0.001).

### Treatment with JNJ-47965567 reduced body weight loss in female SOD1^G93A^ mice

To study body weight loss – a marker of disease progression in ALS and of muscle loss (Ludolph et al., 2010; Cervetto et al., 2013) – mice included in the study were weighed 4 times per week, as detailed in the Materials and Methods.

**Fig. 4.**
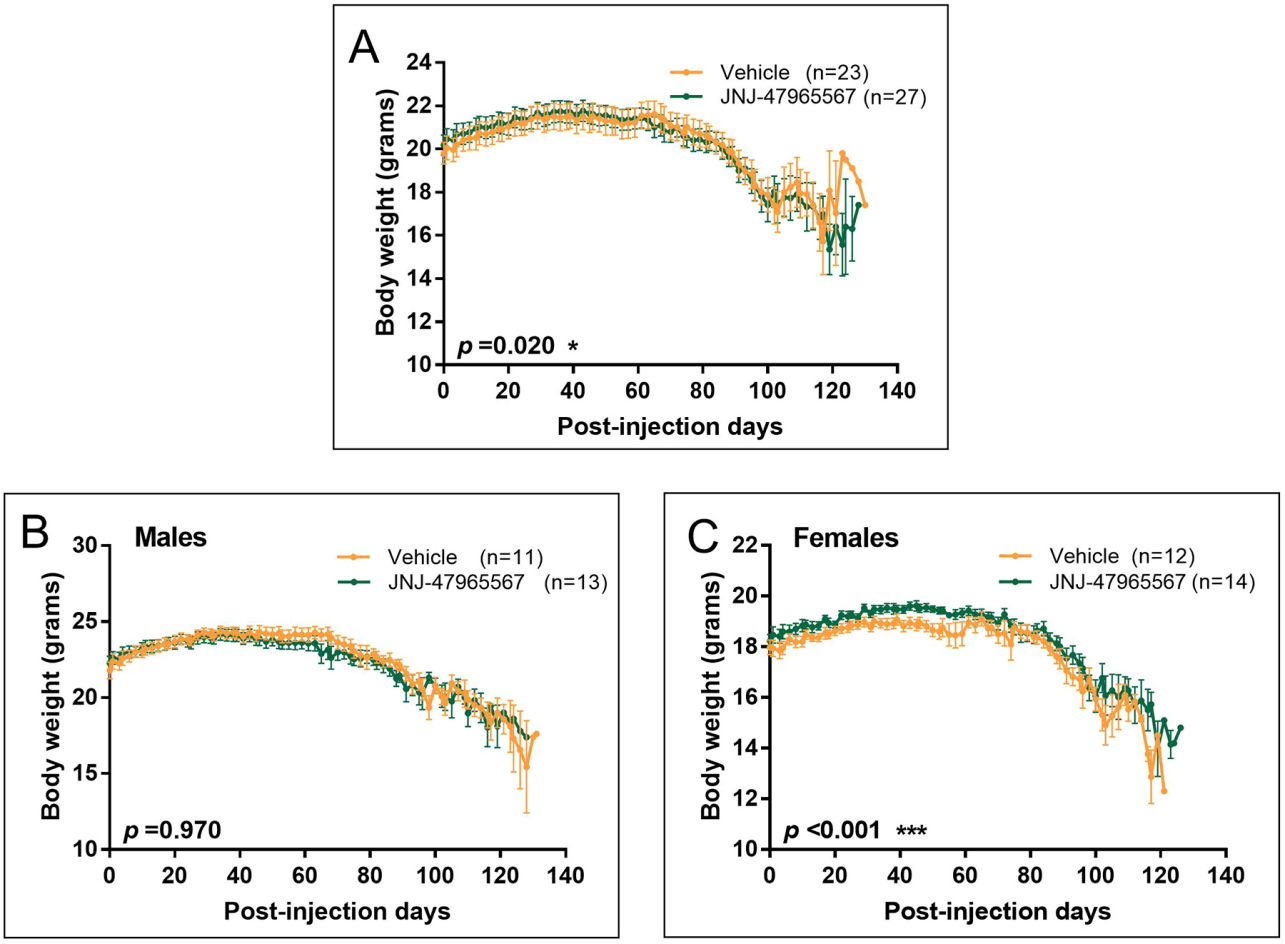
**Variation in body weight of SOD1^G93A^ mice during the treatment.** Vehicle-treated mice are shown in orange; JNJ-47965567-treated mice are represented in green. (A) Body weight variation in the whole group of mice. (B) Body weight variation in the subset of male mice. (C) Body weight variation in the subset of female mice. Data are presented as mean±s.e.m. Data analysis was performed with unpaired *t*-test or Mann–Whitney test, depending on the normality of the data, and differences were considered statistically significant at *P*<0.05.

As shown in Fig. 4A, JNJ-47965567 was able to reduce body weight loss in the whole set of animals (*P*=0.02). However, no difference regarding body weight was observed when treated males were analyzed independently (Fig. 4B; *P*=0.97). In line with the results presented for the disease onset, treatment with JNJ-47965567 significantly reduced body weight loss in females (Fig. 4C; *P*<0.001).

### Treatment with JNJ-47965567 improved motor performance in female SOD1^G93A^ mice

Having observed a delayed disease onset and a milder weight loss, it was expected that JNJ-47965567-treated mice would exhibit a better motor performance in comparison with vehicle-treated mice. As previously detailed, motor performance was evaluated twice per week with the RotaRod apparatus, which measures motor coordination, and 4 times per week with the neurological test NeuroScore.

**Fig. 5.**
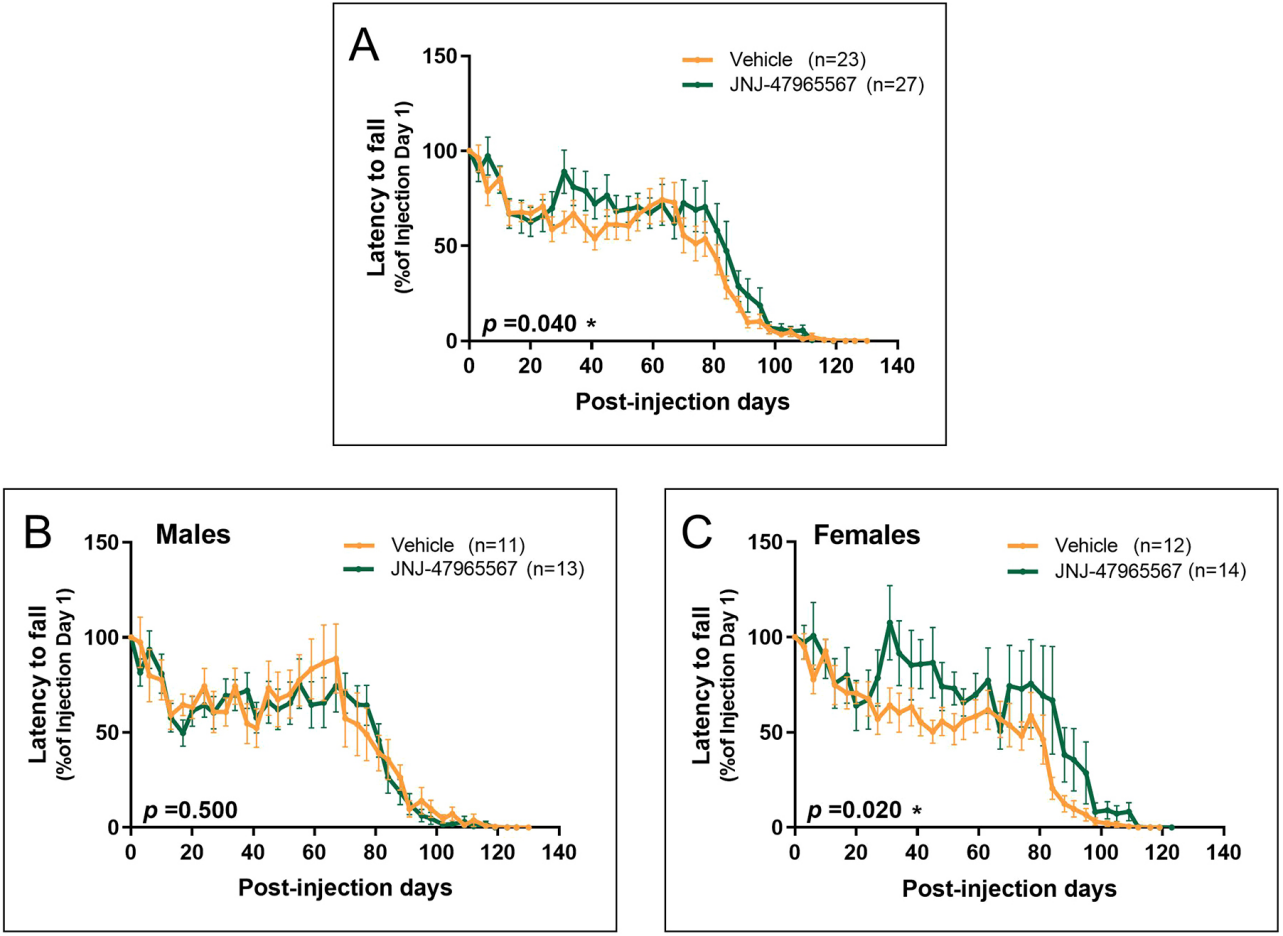
**Evaluation of motor coordination by using the RotaRod apparatus.** Latency to fall on each time-point is presented as the percentage of the latency of the first day, considered as maximum. Mice administered with vehicle are shown in orange; JNJ-47965567-treated mice are represented in green. (A) Variation in latency to fall in the whole group of mice. (B) Variation in latency to fall in the subset of males. (C) Variation in latency to fall in the subset of females. Data are presented as mean±s.e.m. The analysis was performed with unpaired *t*-test or Mann–Whitney test, depending on data normality, and differences were considered statistically significant at *P*<0.05.

Results obtained by using the RotaRod are shown in Fig. 5, expressed as the latency to fall (in seconds) and normalized as the percentage of the values obtained the first day of treatment. Similar to results regarding disease onset and body weight, the RotaRod test revealed differences between vehicle and JNJ-47965567-treated mice. The combined analysis of males and females revealed a higher latency time in treated mice (Fig. 5A; *P*=0.04). Such difference was lost when separately analyzing male data (Fig. 5B; *P*=0.50), and reappeared more clearly when female data were analyzed separately (Fig. 5C; *P*=0.02).

**Fig. 6.**
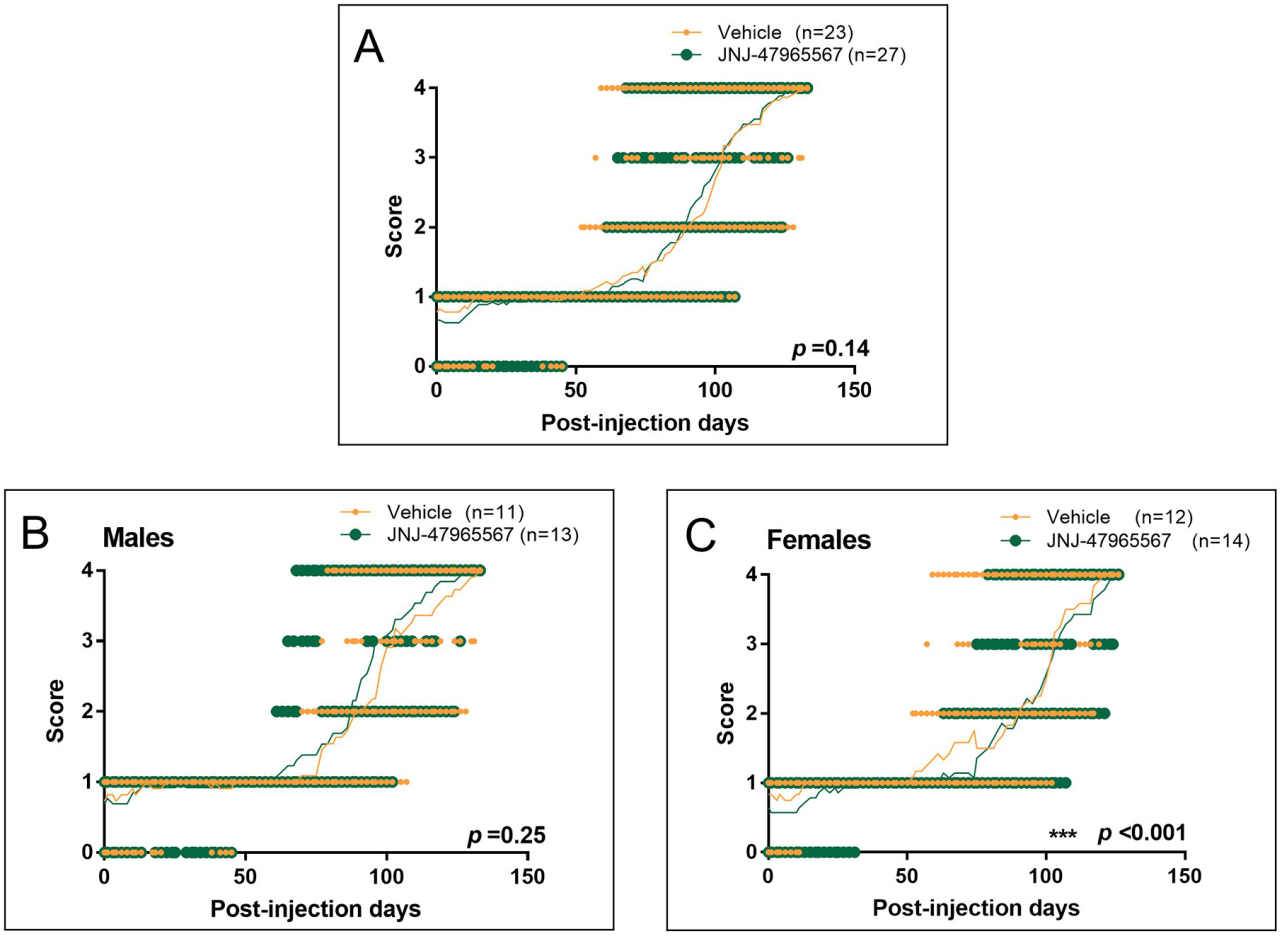
**Disease progression evaluated according to the NeuroScore test, ranging from 0 (i.e. presymptomatic) to 4 (i.e. endpoint).** Graphs show individual scores over the duration of the disease; the mean is represented by a thin line. Mice administered with vehicle are shown in orange; JNJ-47965567-treated mice are represented in green. (A) NeuroScore variation in the whole group of mice. (B) NeuroScore variation in the subset of male mice. (C) NeuroScore variation in the subset of female mice. Data analysis was performed with unpaired *t*-test or Mann–Whitney test, depending on data normality, and differences were considered statistically significant at *P*<0.05.

Similar results were observed for the phenotypic test NeuroScore, which measures disease progression (Fig. 6). In this case, no differences were observed when males and females were analyzed together (Fig. 6A; *P*=0.14), nor when males were independently considered (Fig. 6B; *P*=0.25). However – and following the trend observed in disease onset, weight loss and motor coordination – treatment significantly delayed disease progression in the female subset of animals, as shown in Fig. 6C (*P*<0.001).

### Treatment with JNJ-47965567 did not affect the survival of SOD1^G93A^ mice

To conclude the analysis of the clinical effects of the treatment, we wondered whether the chronic administration of JNJ-47965567 can extend the lifespan of the animals. For this matter, survival was defined by the number of days elapsed until animals reached the humane endpoint of the study or a NeuroScore (NS) 4 (see Materials and Methods).

**Fig. 7.**
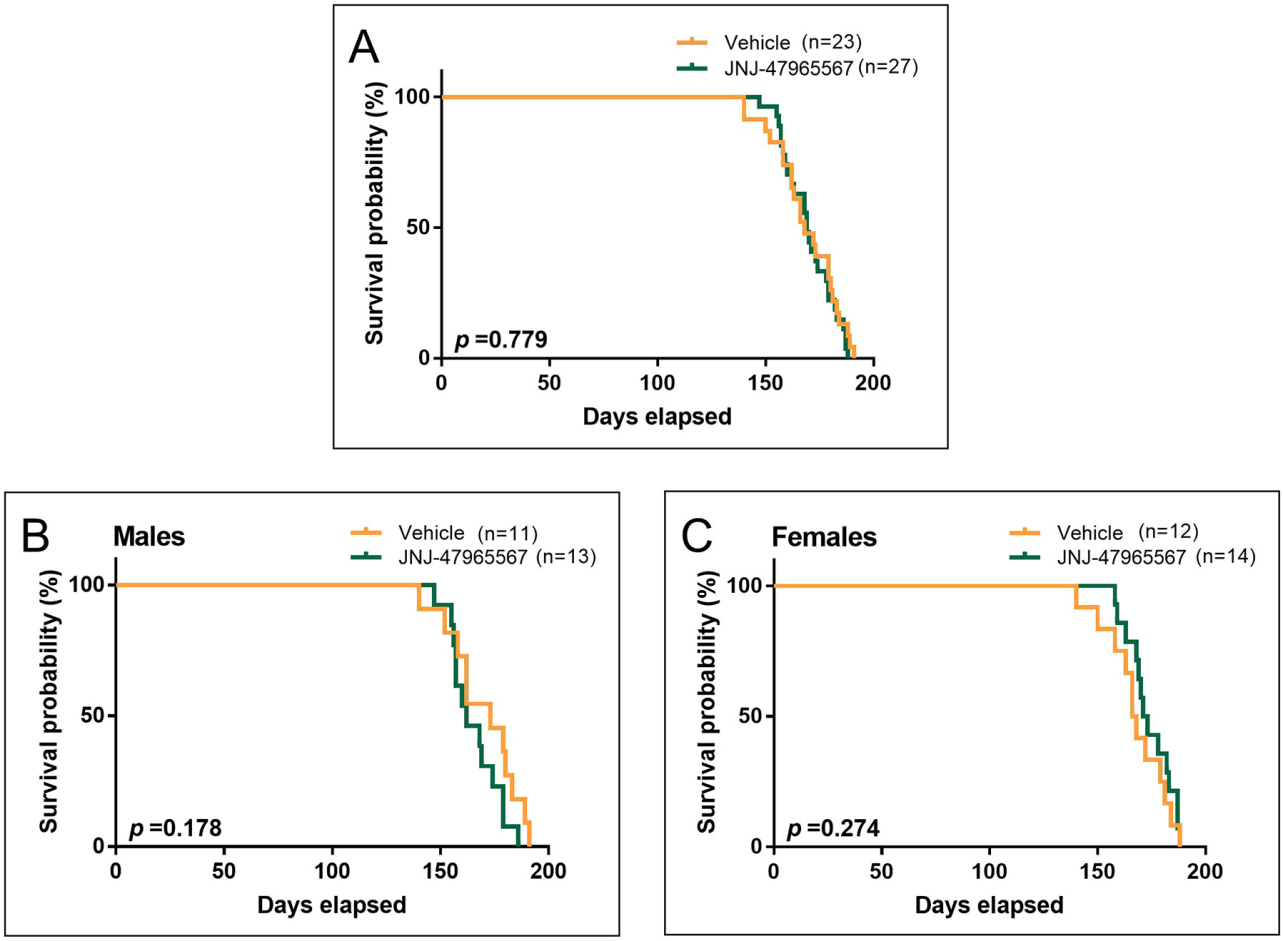
**Survival probability of SOD1^G93A^ ALS mice administered i.p. 4 times per week with vehicle (in orange) or 30 mg/kg JNJ-47965567 (in green).** Survival endpoint was defined as the time mice were unable to right themselves after being placed on either body side. (A) Kaplan–Meier plot showing survival probability in the whole group of mice. (B) Kaplan–Meier plot showing survival probability in the subset of male mice. (C) Kaplan–Meier plot showing survival probability in the subset of female mice. Data analysis was performed using Cox regression, introducing age at first injection as a co-variable. Differences were considered statistically significant at *P*<0.05.

Kaplan–Meier survival curves are plotted in Fig. 7. The combined analysis of males and females showed no difference in survival between vehicle- and JNJ-47965567-treated mice (Fig. 7A; 168.9±3.1 for vehicle- and 169.8±2.2 for JNJ-47965567-treated mice, *P*=0.779). In the case of males, vehicle-treated mice showed a tendency to live longer than those administered with JNJ-47965567, although this difference was not statistically significant (Fig. 7B; 169.9±4.9 for vehicle- and 165.3±3.2 for JNJ-47965567-treated mice, *P*=0.178). The opposite occurred with females treated with JNJ-47965567; these tended to live longer than vehicle-treated mice, although no statistical difference was observed (Fig. 7C; 167.9±4.1 for vehicle- and 174.0±2.8 for JNJ-47965567-treated mice, *P*=0.274).

### Treatment with JNJ-47965567 did not improve motor neuron viability of endpoint SOD1^G93A^ mice

**Fig. 8.**
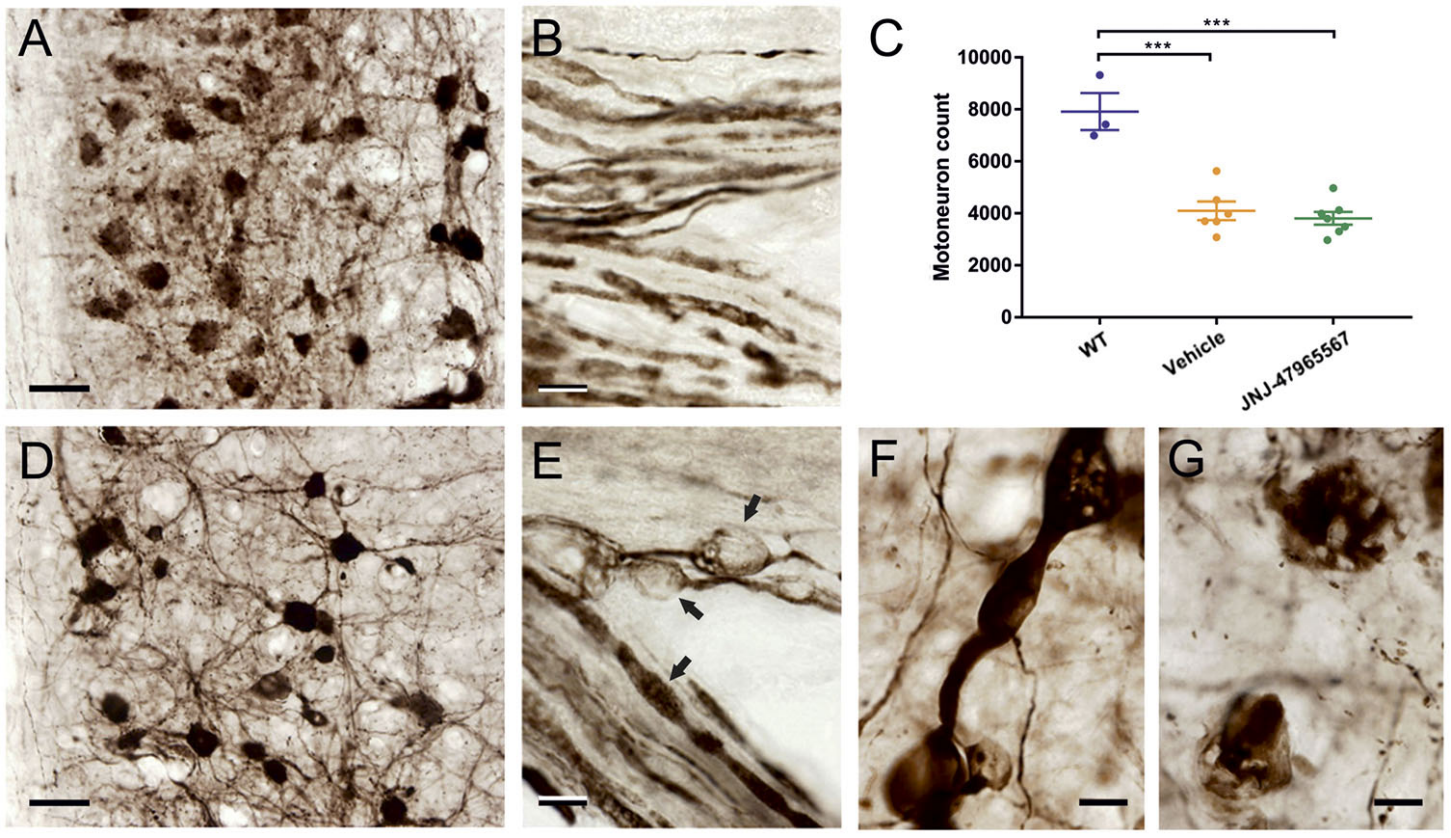
**ChAT immunolabeling of WT and SOD1^G93A^ mice at endpoint stage.** (A,B,D-G) Staining of spinal cords for ChAT reveals a reduction in the total number of motor neurons, together with degenerative changes in cell bodies and neurites in vehicle-treated SOD1^G93A^ ALS mice (D-G) compared with age-matched wild-type animals (A,B). (C) Decrease in number approached a highly significant 50% in both vehicle- and JNJ-47965567-treated animals of mixed sexes (*n*=6). Data are shown as mean±s.e.m. (E) Motor axons exiting the ventral horn in the ventral root show abundant swellings and probable vacuolations (arrows in E). (F) Similar dilatations are often found in thickened dendrites. Moreover, ChAT-positive profiles, probably representing remnants of neuronal perikarya (G), intermingle with a modest number of large neurons that show strong ChAT labeling (D). Scale bars: 50 µm (A,D); 10 µm (B,E-G).

Next, we wondered whether JNJ-47965567 treatment was able to increase motor neuron survival in the spinal cord of SOD1^G93A^ mice. Cell bodies of neurons that stained positively for choline acetyltransferase (ChAT) were considered to be and counted as motor neurons. Treatment with JNJ-47965567 was unable to reduce motor neuron loss in the analyzed mixed sex group of animals of *P*=0.816, *n*=6 at the humane endpoint of the study (Fig. 8C). However, the reduction in the number of motor neurons was evident and significant when comparing WT individuals and SOD1^G93A^ mice (*P*<0.001). Clear morphological abnormalities were found in endpoint SOD1^G93A^ mice (Fig. 8D-G), which were not present in age-matched WT animals (Fig. 8A,B). A lower number of motor neurons, some of which showed clear signs of degeneration, was present in ALS animals (Fig. 8A,D,G); also observed were vacuolation and/or beading and swelling of neuronal processes (Fig. 8B,E).

### Treatment with JNJ-47965567 did not modify protein expression of endpoint SOD1^G93A^ mice

To assess whether treatment was diminishing microglial activation and proliferation, we studied the expression of the microglial marker allograft inflammatory factor 1 (AIF1, hereafter referred to as Iba-1). Likewise, we then determined whether the administration increased motor neuron survival by analyzing expression of the motor neuron marker ChAT. Last, we checked whether P2X7R expression or the levels of mutated human SOD1 were affected by the treatment. These four parameters were analyzed in endpoint female SOD1^G93A^ mice by western blotting. No differences were observed between vehicle- and JNJ-47965567-treated females regarding the expression of any of the analyzed proteins (*n*=3, Fig. S1).

## DISCUSSION AND CONCLUSIONS

In this study, we analyzed the effects that chronic administration of the selective, potent and brain-penetrant P2X7R antagonist JNJ-47965567 has on the SOD1^G93A^ mouse model of ALS. Administration of the antagonist was able to delay disease onset, reduce body weight loss and improve motor performance in female individuals, although no effect was observed in males. No differences were observed regarding motor neuron survival and protein expression at the humane endpoint in animals of a mixed sex group.

Our results are generally in line with previously published data, although it is crucial to highlight the differences found between the studies, which are summarized in Table 1. First published studies assessed the effect of chronic administration of the antagonist BBG. Cervetto and colleagues Cervetto et al., (2013) reported improved motor performance in both sexes, which in this case, was found more predominant in males when compound administration started at P90 or pre-onset. In contrast to our results, reduced weight loss was also reported in the subgroup of males but not in females. The administration of BBG also delayed disease onset in mice when administered from P100 onwards or late pre-onset, but no differences between sexes were observed (Apolloni et al., 2014). A reduction in body weight loss, accompanied by prolonged survival was found in females when BBG treatment started at P60 or pre-onset, although disease onset and motor performance were not modified (Bartlett et al., 2017). Even though these results point to a possible implication of P2X7R in ALS progression, their disparity is striking. These discrepancies could be due to the differences in dosing, starting-point of administration and the use of BBG itself. Although BBG is widely used *in vitro* and *in vivo* as a P2X7R antagonist, it is not selective for this receptor, as it is known to target P2X1R (Seyffert et al., 2004), P2X5R (Bo et al., 2003), P2X4R, voltage-gated sodium channels (Jo and Bean, 2011), as well as the ATP-release channel pannexin-1 (Qiu and Dahl, 2009). Therefore, there is no clear evidence that the benefits of BBG treatment are only due to P2X7R inhibition; they might be a consequence of targeting any of the other routes. However, our results can be directly linked to the blockade of P2X7R, as JNJ-47965567 is a very potent and CNS-permeable antagonist, with very high selectivity for P2X7R, as well as high target engagement and efficacy at the dose used. Moreover, its way of administration has already been demonstrated (Bhattacharya et al., 2013).

However, a recently published article that described the chronic administration of JNJ-47965567 to SOD1^G93A^ mice did not report any beneficial outcomes (Ly et al., 2020). The differences between the above mentioned study and our study here is that Ly and colleagues performed compound administration 3 times per week from onset or P100, whereas in our current study, mice received antagonist injections 4 times per week at pre-symptomatic stage or P60 onwards. The very surprising difference in results between the two studies reveals the great importance of study design, and suggests that a constant and consistent blockade of the P2X7R is crucial for the therapeutic effect to take place in SOD1^G93A^ mice. It has been shown that JNJ-47965567 has a residence time in the CNS of ∼2-4 h after a 30 mg/kg i.p. dose (Jimenez-Pacheco et al., 2016). Thus, administration of the compound 4 times per week might be better in order to maintain specific drug concentrations in the CNS and the antagonism of the receptor. Therefore, an even more frequent dosage, such as every 24 h, or the use of a different antagonist with longer residence time in the CNS, could be more suitable for the blockade of P2X7R and treatment of SOD1^G93A^ mice.

Another experiment showing the importance of study design and the selection of the antagonist is that of the chronic administration of A-804598 – a CNS-permeable, potent and selective antagonist – to SOD1^G93A^ mice from P100 or at pre-onset, 5 times per week. In contrast to our results, this treatment did not exert any beneficial effect (Fabbrizio et al., 2017). As the potencies of JNJ-47965567 and A-804598 are similar and since they have the same binding site, the discrepancy in these results could be due to the reduced brain-to-plasma ratio of the latter compared with that of JNJ-47965567 (Bhattacharya et al., 2013; Karasawa and Kawate, 2016). Differences could also be due to the distinct starting point of administration as, in this case, the administration was performed at late pre-onset stages of the disease.

Owing to the high heterogeneity of treatment starting-points between the aforementioned works, drawing conclusions about the best time to start the treatment is challenging. However, it is evident that treatment start points substantially influence study outcomes. These differences could be due to the disparate activation states developed by microglia in response to P2X7R activation. Microglia are highly dynamic and plastic cells that are extremely sensitive to the smallest perturbation and that undergo irreversible imprinting towards anti-inflammatory (M2) or pro-inflammatory (M1) phenotypes (Walker and Lue, 2015; Parisi et al., 2016; Tang and Le, 2016). A recent study has shown that treatment with the P2X7R agonist BzATP at late pre-onset or P105 helps to preserve neuromuscular junction morphology, and delays denervation and atrophy in SOD1^G93A^ mice (Fabbrizio et al., 2020). Moreover, ablation of P2X7 receptor in SOD1^G93A^ mice exerted a detrimental effect in disease progression and increased pro-inflammatory markers (Apolloni et al., 2013a). Therefore, to find biomarkers that can reflect the activation state of microglia during ALS progression would help to determine the best time to start treatment. Also, due to the differences between mice strains of individual laboratories, i.e. different phenotypic symptoms at variable postnatal days (see Table 1), it would be very useful for future works to specifically explain the state of the animals when treatment commences; that is not only as ‘days after birth’ but also by defining symptoms. Especially the latter can more easily be related to the individual state of each animal and could reduce heterogeneity.

Another remarkable outcome of our study is the sex difference in relation to treatment, which has been pointed out previously (Cervetto et al., 2013; Bartlett et al., 2017). This sex-specific effect could be due to the contribution of sex hormones, known to play a key role in metabolism (Bame et al., 2012). The implication of hormones could be further assessed by using our experimental set-up but with ovariectomized or castrated animals. However, these differences could also be due to the use of the SOD1^G93A^ model itself, as differences in terms of disease progression, onset and survival have been reported in this mouse strain, as well as in patients carrying mutations of SOD1 (Pfohl et al., 2015; Tang et al., 2019). Therefore, to confirm the validity of the results observed with our model, the experiment could be repeated by using different transgenic mouse models, e.g. TDP-43^A315T^ (White et al., 2018) or h(G_4_C_2_)_37-500_ (Liu et al., 2016), carrying mutations in different proteins [TAR DNA-binding protein 43 (TARDBP) or guanine nucleotide exchange C9orf72 (CI072), respectively].

Even though some differences in molecular outcomes after treatment with BBG have been reported, such as reduced microgliosis, and decreased inflammatory markers and motor neuron loss (Apolloni et al., 2014), we did not observe any of these differences between JNJ-47965567-treated and vehicle-treated mice. The lack of difference in terms of protein expression is in line with the differences observed when treating 3 times per week with JNJ-47965567 (Ly et al., 2020). However, in our case, it could also be explained by the reduced number of animals analyzed (*n*=3), as a higher number of mice are needed to yield stronger conclusions. As initially expected given the experimental design, we found no difference between treatments regarding the number of motor neurons. As all tissue samples were taken at the humane endpoint, we had expected that all the animals would reach the same level of degeneration, independently of treatment duration or type of compound administered. Therefore, it is possible that our treatment routine yielded a mild beneficial effect regarding clinical outcome, which is not necessarily reflected in motor neuron survival or protein expression. In this regard, a protocol according to which all animals were euthanized at the same pre-defined post-injection day could help to discern the effect of P2X7R antagonism at the molecular level and in terms of motor neuron survival.

In conclusion, the current study demonstrates that chronic administration of the P2X7R antagonist JNJ-47965567, 4 times per week from pre-onset until the humane endpoint delayed disease onset, reduced body weight loss and improved motor parameters in female SOD1^G93A^ mice. However, treatment did not show any benefit on males and did not affect motor neuron survival, neither in females nor in males. The outcomes of this study could be useful for the design of new preclinical studies that use P2X7R antagonists with better pharmacokinetic profiles, and to understand the role of this receptor in the pathogenesis and eventual treatment of ALS.

## MATERIALS AND METHODS

### Animals

Mouse experiments were performed with the approval of the Ethics Research Committee of Universidad Autónoma de Madrid (Spain) and according to the code of ethics and guidelines established by European Community Directive (2010/63/EU) and Spanish legislation (RD53/2013). Mice hemizygous for the human SOD1^G93A^ transgene and back-crossed (>5 generations) onto a C57BL/6J background [B6.Cg-Tg (SOD1*G93A) 1Gur/J] were originally provided by Josep E. Esquerda (Universidad de Lérida, Spain).

Mice were bred and maintained at the Animal Facility of the Medical School, Universidad Autónoma de Madrid, Madrid (Spain). At weaning, transgenic SOD1^G93A^ mice were genotyped as previously described (Apolloni et al., 2013a) and housed in a temperature-controlled environment on a 12:12 h light-dark cycle. SOD1^G93A^ mice were matched for age and sex, and randomly divided into two treatment groups, JNJ-47965567 (Fig. 1) and vehicle. They were caged with paired littermates, with 4-5 females or 2-3 males per cage (unless fighting required separation). Food and water were available *ad libitum*. The development of symptoms was closely monitored using the neurological NeuroScore (NS) test that classifies mice from 0 (i.e. pre-symptomatic) to 4 (endpoint state) (Hatzipetros et al., 2015). When mice became symptomatic and were unable to access water and food (NS 2), longer sippers were placed onto water bottles and food pellets placed directly onto the cage floor.

### Administration of JNJ-47965567 and vehicle

JNJ-47965567 was provided by Janssen Pharmaceutica NV (Beerse, Belgium) and was prepared at a concentration of 5 mg/ml in 30% SBE-β-cyclodextrin. SBE-β-cyclodextrin was first provided by Janssen Pharmaceutica NV (Beerse, Belgium) and then purchased from Cydex Pharmaceuticals. To prepare the solution, the compound was weighed, dissolved in 2 equivalents of HCl (37%) and further diluted in SBE-β-cyclodextrin and adjusted to pH 4-4.5. Both solution and vehicle (30% SBE-β-cyclodextrin) were filter sterilized through 0.22 μm filters (Merck-Millipore) and used within one week. Mice were injected intraperitoneally (i.p.) 4 times per week (evenly distributed during the week), starting at pre-symptomatic stage or P60 (ranging from P57 to P78) until endpoint (i.e. NS 4 or the inability of mice to right themselves within the first 10 s after being placed on either of their sides). This endpoint occurred at approximately P160, concordant with the average life expectancy of SOD1^G93A^ mice in a C57BL/6J background (157.1±9.3 days, The Jackson Laboratory) (Wooley et al., 2005). Injections were administered to alternating sides of the abdomen to minimize irritation, and administration was blinded for the investigator carrying out injections and analysis.

### Body weight measurements

Body weight was measured 4 times per week (evenly distributed during the week), prior to each injection. Measurements were made at the same time each day to minimize variation due to diurnal changes in food intake and exercise. Body weight was used to assess disease onset, defined as the day the animal was losing weight for 3 consecutive days without recovery observed, corresponding to the body weight peak (Miana-Mena et al., 2005; Leitner et al., 2009).

### RotaRod performance

Motor coordination was measured with the RotaRod apparatus (Stoelting Co.). The test, which measures the time mice are able to walk on a rotating cylinder at increasing speed, was performed twice per week (evenly spread during the week) and started with the start of treatment. Initial speed was 8 rpm, increased by 1 rpm every 8 s up to a maximum of 120 s. Before treatment start, mice were trained for 3 consecutive days, with 5 repetitions each day. Once trained, time spent walking on the RotaRod was annotated and the value was the mean of three repetitions. As motor coordination is highly variable between mice, the time spent on the RotaRod on the first day of injection was considered as 100% for each individual; later values were expressed as percentage of the initial value.

### NeuroScore

As previously mentioned, the NeuroScore test (Hatzipetros et al., 2015) was used to assess disease progression and neurological deficit in SOD1^G93A^ mice along the study. The test was performed 4 times per week (evenly distributed during the week), prior to the injection of that day. Mice were considered as pre-symptomatic, i.e. NS 0, when no tremor was observed in the hindlimbs and they were able to walk normally. When tremor appeared in the hindlimbs or these partially collapsed, animals were scored at NS 1. When toes curled downwards at least twice during a 75 cm walk, mice were considered to be at NS 2. NS 3 implied rigid paralysis or minimal joint movement in the hindlimbs, which were not used for forward motion. Lastly, animals were considered to be at NS 4 when no forward motion was observed and they were unable to right themselves within the first 10 s after being placed on either of their sides. The NS 4 was considered to be the humane endpoint of the study and considered for survival analysis. A summary of the study design is shown in Fig. 2.

### Tissue preparation and immunostaining

At the humane endpoint, a subset of mice was anesthetized (Dolethal, 50 mg/kg i.p.) and perfused through the ascending aorta with 0.9% NaCl followed by 4% paraformaldehyde (PFA) in 0.1 M phosphate buffer (PB). Wild-type age-matched mice were euthanized following the same protocol to be used as controls. The caudal part of the spinal cord was excised by dissecting it between vertebrae T9-T10 and the cauda equina. The rostralmost 3 mm were cut off and, again, a transection was carried out 11 mm caudalwards. The piece obtained, which had in its center the lumbar enlargement (segments L2-L6) (Harrison et al., 2013) was postfixed in 4% buffered PFA for 24 h and cryoprotected for 2 days in 30% sucrose in 0.1 M PB. The spinal blocks were frozen at oblique angles over the horizontal, and serially cut at 40 µm in a sliding microtome (Leica SM2400, Leica Biosystems, Nussloch, Germany). Every fourth section was processed for free-floating immunostaining. After several washes in 0.1 M PB, inactivation of endogenous peroxidase with 1% H_2_O_2_ in PB and preincubation in a blocking solution with PBS, rabbit serum and 2% Triton X-100 for 1 h, the sections were incubated with 1:100 anti-ChAT (goat, RRID: AB_2079751, Cat# AB144P, Sigma-Aldrich), overnight at 4°C in agitation. This was followed by a 2-h incubation in biotinylated secondary antibody 1:500 anti-goat (rabbit, RRID: AB_2336126, Cat# BA-5000, Vector Laboratories). Sections were then incubated in avidin–biotin solution (VECTASTAIN^®^ Elite^®^ ABC HRP Kit, #PK-6100, Vector Laboratories) in 0.02 M PBS with 2% Triton X-100 and revealed in 0.05% 3,3′-diaminobenzidine (DAB) in 0.1 M PB, with 0.008% cobalt chloride, 0.0064% nickel sulfate and 0.001% H_2_O_2_ added. Finally, sections were mounted on glass slides, dehydrated, defatted and mounted on coverslips using DePeX.

### Microscopy and stereology

Spinal cord sections were examined and photographed using an Olympus BX61 microscope. The total number of motor neurons was estimated using the optical fractionator procedure (West et al., 1991) and ChAT-immunoreactive cell bodies in the ventral horn as counting units. This analysis was performed in an integrated stereological setup that included an Olympus BX61 microscope with a high-precision motorized microscope stage (Prior Proscan II, Prior Scientific Inc., Rockland, MA, USA), a 0.3-µm resolution microcator and an Olympus DP71 camera. The control of the stage movements and the interactive test grids were provided by the NewCast stereological software (ver. 4.6.1.630, Visiopharm, Hørsholm, Denmark). A plan apochromatic 100× oil immersion lens (1.40 NA, UPlanSApo, Olympus) was used for neuron counting and for estimating section thickness at each sampling point. On average, 73 neurons were counted (ranging from 45 to 146). The precision of the estimates, determined by estimating the coefficient of error (CE) as described for systematic uniform random samples (Gundersen et al., 1999), was between 0.09 and 0.19 (mean=0.13). The investigator performing stereological procedures was blinded to the treatment assigned to each case.

### Western blots

At the humane endpoint, a subset of mice was anesthetized (Dolethal, 50 mg/kg i.p.) and perfused through the ascending aorta with 0.9% NaCl. Non-treated SOD1^G93A^ mice were euthanized at humane endpoint to be used as controls. Spinal cord was dissected between vertebrae T9-T10 and the cauda equina, and stored at −80°C. Proteins were extracted by sonication in RIPA buffer with protease inhibitors and quantified using the Pierce™ BCA protein assay kit (#23227, ThermoFisher Scientific). 12% polyacrylamide gels were casted for Iba-1 and hSOD1 analysis and 8% gels for ChAT and P2X7R quantification. 30 μg of protein was loaded from each sample, transferred to polyvinylidene fluoride (PVDF) membranes (Immobilon-P, Merk Millipore), blocked for 1 h with 5% milk in TBST at room temperature and incubated overnight with the following primary antibodies diluted in 3.5% milk in TBST: 1:1000 anti-Iba-1 (rabbit, RRID: AB_839504, Cat# 019-19741, FUJIFILM Wako Chemicals), anti-P2X7R (1:200, rabbit, RRID: AB_2040068, Cat# APR-004, Alomone Labs), anti-ChAT (1:500, goat, RRID: AB_2079751, Cat# AB144P, Sigma-Aldrich), anti-hSOD1 (1:500, mouse, RRID:AB_10015296, Cat# MM-0070-2-P, MediMabs), anti-β-actin (1:50000, mouse, peroxidase-conjugated, RRID: AB_262011, Cat# A3854, Sigma-Aldrich). Membranes were washed with TBST and incubated for 1 h at room temperature with the corresponding secondary antibodies diluted in 3.5% milk in TBST (1:5000, goat anti-rabbit IgG-HRP, RRID: AB_631746, Cat# sc-2004, Santa Cruz Biotechnology; 1:5000, donkey anti-goat IgG-HRP, RRID: AB_631728, Cat# sc-2020, Santa Cruz Biotechnology, 1:5000 goat anti-mouse IgG-HRP, RRID: AB_631737, Cat# sc-2031, Santa Cruz Biotechnology). Membranes were washed with TBST, incubated with Clarity Western ECL substrate (#1705061, BioRad) and chemiluminescence was detected with the ChemiDoc™ XRS+ System (#1708265, BioRad).

### Data and statistical analysis

For survival and disease onset analysis, Cox regression test was performed, introducing a correction for the variation on age at first injection. Disease onset was considered as the day animals were losing weight for the third day in a row without recovery.

For weight variation, RotaRod and NeuroScore data, D'Agostino–Pearson test was applied to analyze data normality. If data followed a normal distribution, an unpaired Student's *t*-test comparing the mean of data points of vehicle and treatment was used. If data did not follow a normal distribution, the non-parametric Mann–Whitney test was used to compare the two groups.

For motor neuron number and western blot analysis, D'Agostino–Pearson test was applied to analyze the normality of the data, and normal data were analyzed with ordinary one-way ANOVA and post hoc Tukey's multiple comparisons test.

Data were analyzed with GraphPad Prism 7 and IBM SPSS Statistics. Statistical differences with *P*<0.05 were considered significant. The data and statistical analyses comply with the recommendations on experimental design and analysis in pharmacology (Curtis et al., 2018).

## Supplementary Material

Supplementary information

## References

[DMM045732C1] Apolloni S., Amadio S., Montilli C., Volonte C. and D'ambrosi N. (2013a). Ablation of P2X7 receptor exacerbates gliosis and motoneuron death in the SOD1-G93A mouse model of amyotrophic lateral sclerosis. *Hum. Mol. Genet.* 22, 4102-4116. 10.1093/hmg/ddt259 23736299

[DMM045732C2] Apolloni S., Parisi C., Pesaresi M. G., Rossi S., Carrì M. T., Cozzolino M., Volonté C. and D'Ambrosi N. (2013b). The NADPH oxidase pathway is dysregulated by the P2X7 receptor in the SOD1-G93A microglia model of amyotrophic lateral sclerosis. *J. Immunol.* 190, 5187-5195. 10.4049/jimmunol.1203262 23589615

[DMM045732C3] Apolloni S., Amadio S., Parisi C., Matteucci A., Potenza R. L., Armida M., Popoli P., D'ambrosi N. and Volonte C. (2014). Spinal cord pathology is ameliorated by P2X7 antagonism in a SOD1-mutant mouse model of amyotrophic lateral sclerosis. *Dis. Model. Mech.* 7, 1101-1109. 10.1242/dmm.017038 25038061 PMC4142730

[DMM045732C4] Bame M., Pentiak P. A., Needleman R. and Brusilow W. S. A. (2012). Effect of sex on lifespan, disease progression, and the response to methionine sulfoximine in the SOD1 G93A mouse model for ALS. *Gend. Med.* 9, 524-535. 10.1016/j.genm.2012.10.014 23217569

[DMM045732C5] Bartlett R., Sluyter V., Watson D., Sluyter R. and Yerbury J. J. (2017). P2X7 antagonism using Brilliant Blue G reduces body weight loss and prolongs survival in female SOD1^G93A^ amyotrophic lateral sclerosis mice. *PeerJ* 5, e3064 10.7717/peerj.3064 28265522 PMC5335685

[DMM045732C6] Bhattacharya A., Wang Q., Ao H., Shoblock J. R., Lord B., Aluisio L., Fraser I., Nepomuceno D., Neff R. A., Welty N. et al. (2013). Pharmacological characterization of a novel centrally permeable P2X7 receptor antagonist: JNJ-47965567. *Br. J. Pharmacol.* 170, 624-640. 10.1111/bph.12314 23889535 PMC3792000

[DMM045732C7] Bo X., Jiang L.-H., Wilson H. L., Kim M., Burnstock G., Surprenant A. and North R. A. (2003). Pharmacological and biophysical properties of the human P2X_5_ receptor. *Mol. Pharmacol.* 63, 1407-1416. 10.1124/mol.63.6.1407 12761352

[DMM045732C8] Bonafede R. and Mariotti R. (2017). ALS pathogenesis and therapeutic approaches: the role of mesenchymal stem cells and extracellular vesicles. *Front. Cell. Neurosci.* 11, 80 10.3389/fncel.2017.00080 28377696 PMC5359305

[DMM045732C9] Brites D. and Vaz A. R. (2014). Microglia centered pathogenesis in ALS: insights in cell interconnectivity. *Front. Cell. Neurosci.* 8, 117 10.3389/fncel.2014.00117 24904276 PMC4033073

[DMM045732C10] Calvo A. C., Manzano R., Mendonça D. M. F., Muñoz M.­J., Zaragoza P. and Osta R. (2014). Amyotrophic lateral sclerosis: a focus on disease progression. *Biomed Res. Int.* 2014, 925101 10.1155/2014/925101 25157374 PMC4137497

[DMM045732C11] Cervetto C., Frattaroli D., Maura G. and Marcoli M. (2013). Motor neuron dysfunction in a mouse model of ALS: gender-dependent effect of P2X7 antagonism. *Toxicology* 311, 69-77. 10.1016/j.tox.2013.04.004 23583883

[DMM045732C12] Corcia P., Tauber C., Vercoullie J., Arlicot N., Prunier C., Praline J., Nicolas G., Venel Y., Hommet C., Baulieu J.-L. et al. (2012). Molecular imaging of microglial activation in amyotrophic lateral sclerosis. *PLoS ONE* 7, e52941 10.1371/journal.pone.0052941 23300829 PMC3534121

[DMM045732C13] Cruz M. P. (2018). Edaravone (Radicava): a novel neuroprotective agent for the treatment of amyotrophic lateral sclerosis. *P. T.* 43, 25-28. 29290672 PMC5737249

[DMM045732C14] Curtis M. J., Alexander S., Cirino G., Docherty J. R., George C. H., Giembycz M. A., Hoyer D., Insel P. A., Izzo A. A., Ji Y. et al. (2018). Experimental design and analysis and their reporting II: updated and simplified guidance for authors and peer reviewers. *Br. J. Pharmacol.* 175, 987-993. 10.1111/bph.14153 29520785 PMC5843711

[DMM045732C15] Disabato D. J., Quan N. and Godbout J. P. (2016). Neuroinflammation: the devil is in the details. *J. Neurochem.* 139 Suppl. 2, 136-153. 10.1111/jnc.13607 26990767 PMC5025335

[DMM045732C16] Doble A. (1996). The pharmacology and mechanism of action of riluzole. *Neurology* 47, S233-S241. 10.1212/WNL.47.6_Suppl_4.233S 8959995

[DMM045732C17] Fabbrizio P., Amadio S., Apolloni S. and Volonté C. (2017). P2X7 receptor activation modulates autophagy in SOD1-G93A mouse microglia. *Front. Cell. Neurosci.* 11, 249 10.3389/fncel.2017.00249 28871219 PMC5566572

[DMM045732C18] Fabbrizio P., Apolloni S., Bianchi A., Salvatori I., Valle C., Lanzuolo C., Bendotti C., Nardo G. and Volonté C. (2020). P2X7 activation enhances skeletal muscle metabolism and regeneration in SOD1G93A mouse model of amyotrophic lateral sclerosis. *Brain Pathol.* 30, 272-282. 10.1111/bpa.12774 31376190 PMC7065186

[DMM045732C19] Gandelman M., Peluffo H., Beckman J. S., Cassina P. and Barbeito L. (2010). Extracellular ATP and the P2X7 receptor in astrocyte-mediated motor neuron death: implications for amyotrophic lateral sclerosis. *J. Neuroinflammation* 7, 33 10.1186/1742-2094-7-33 20534165 PMC2901222

[DMM045732C20] Gundersen H. J. G., Jensen E. B. V., Kieu K. and Nielsen J. (1999). The efficiency of systematic sampling in stereology—reconsidered. *J. Microsc.* 193, 199-211. 10.1046/j.1365-2818.1999.00457.x 10348656

[DMM045732C21] Gurney M., Pu H., Chiu A., Dal Canto M., Polchow C., Alexander D., Caliendo J., Hentati A., Kwon Y., Deng H. et al. (1994). Motor neuron degeneration in mice that express a human Cu,Zn superoxide dismutase mutation. *Science* 264, 1772-1775. 10.1126/science.8209258 8209258

[DMM045732C22] Hardiman O., Al-Chalabi A., Chio A., Corr E. M., Logroscino G., Robberecht W., Shaw P. J., Simmons Z. and Van Den Berg L. H. (2017). Amyotrophic lateral sclerosis. *Nat. Rev. Dis. Primers* 3, 17085 10.1038/nrdp.2017.85 29052611

[DMM045732C23] Harrison M., O'Brien A., Adams L., Cowin G., Ruitenberg M. J., Sengul G. and Watson C. (2013). Vertebral landmarks for the identification of spinal cord segments in the mouse. *Neuroimage* 68, 22-29. 10.1016/j.neuroimage.2012.11.048 23246856

[DMM045732C24] Hatzipetros T., Kidd J. D., Moreno A. J., Thompson K., Gill A. and Vieira F. G. (2015). A quick phenotypic neurological scoring system for evaluating disease progression in the SOD1-G93A mouse model of ALS. *J. Vis. Exp.*, 104, 53257 10.3791/53257 PMC4692639 26485052

[DMM045732C25] Jimenez-Pacheco A., Diaz-Hernandez M., Arribas-Blázquez M., Sanz-Rodriguez A., Olivos-Oré L. A., Artalejo A. R., Alves M., Letavic M., Miras-Portugal M. T., Conroy R. M. et al. (2016). Transient P2X7 receptor antagonism produces lasting reductions in spontaneous seizures and gliosis in experimental temporal lobe epilepsy. *J. Neurosci.* 36, 5920-5932. 10.1523/JNEUROSCI.4009-15.2016 27251615 PMC6601816

[DMM045732C26] Jo S. and Bean B. P. (2011). Inhibition of neuronal voltage-gated sodium channels by brilliant blue G. *Mol. Pharmacol.* 80, 247-257. 10.1124/mol.110.070276 21536754 PMC3141889

[DMM045732C27] Karasawa A. and Kawate T. (2016). Structural basis for subtype-specific inhibition of the P2X7 receptor. *eLife* 5, e22153 10.7554/elife.22153 27935479 PMC5176352

[DMM045732C28] Leitner M., Menzies S. and Lutz C. (2009). Working with ALS mice: guidelines for preclinical testing & colony management. *Jackson Lab.*

[DMM045732C29] Liu Y., Pattamatta A., Zu T., Reid T., Bardhi O., Borchelt D. R., Yachnis A. T. and Ranum L. P. W. (2016). C9orf72 BAC mouse model with motor deficits and neurodegenerative features of ALS/FTD. *Neuron* 90, 521-534. 10.1016/j.neuron.2016.04.005 27112499

[DMM045732C30] Ludolph A. C., Bendotti C., Blaugrund E., Chio A., Greensmith L., Loeffler J.-P., Mead R., Niessen H. G., Petri S., Pradat P.-F. et al. (2010). Guidelines for preclinical animal research in ALS/MND: a consensus meeting. *Amyotroph. Lateral Scler.* 11, 38-45. 10.3109/17482960903545334 20184514

[DMM045732C31] Lutz C. (2018). Mouse models of ALS: past, present and future. *Brain Res.* 1693, 1-10. 10.1016/j.brainres.2018.03.024 29577886

[DMM045732C32] Ly D., Dongol A., Cuthbertson P., Guy T. V., Geraghty N. J., Sophocleous R. A., Sin L., Turner B. J., Watson D., Yerbury J. J. et al. (2020). The P2X7 receptor antagonist JNJ-47965567 administered thrice weekly from disease onset does not alter progression of amyotrophic lateral sclerosis in SOD1 ^G93A^ mice. *Purinergic Signal.* 16, 109-122. 10.1007/s11302-020-09692-4 32170537 PMC7166237

[DMM045732C33] Miana-Mena F. J., Muñoz M. J., Yagüe G., Mendez M., Moreno M., Ciriza J., Zaragoza P. and Osta R. (2005). Optimal methods to characterize the G93A mouse model of ALS. *Amyotroph. Lateral Scler.* 6, 55-62. 10.1080/14660820510026162 16036427

[DMM045732C34] Parisi C., Napoli G., Pelegrin P. and Volonté C. (2016). M1 and M2 functional imprinting of primary microglia: role of P2X7 activation and miR-125b. *Mediators Inflamm.* 2016, 2989548 10.1155/2016/2989548 28090150 PMC5206439

[DMM045732C35] Pfohl S. R., Halicek M. T. and Mitchell C. S. (2015). Characterization of the contribution of genetic background and gender to disease progression in the SOD1 G93A mouse model of amyotrophic lateral sclerosis: a meta-analysis. *J. Neuromuscul. Dis.* 2, 137-150. 10.3233/JND-140068 26594635 PMC4652798

[DMM045732C36] Philips T. and Rothstein J. D. (2015). Rodent models of amyotrophic lateral sclerosis. *Curr. Protoc. Pharmacol.* 69, 5.67.1-5.67.21. 10.1002/0471141755.ph0567s69 26344214 PMC4562058

[DMM045732C37] Qiu F. and Dahl G. (2009). A permeant regulating its permeation pore: inhibition of pannexin 1 channels by ATP. *Am. J. Physiol. Cell Physiol.* 296, C250-C255. 10.1152/ajpcell.00433.2008 18945939 PMC2643853

[DMM045732C38] Seyffert C., Schmalzing G. and Markwardt F. (2004). Dissecting individual current components of co-expressed human P2X1 and P2X7 receptors. *Curr. Top. Med. Chem.* 4, 1719-1730. 10.2174/1568026043387160 15579104

[DMM045732C39] Swanson K. V., Deng M. and Ting J. P.-Y. (2019). The NLRP3 inflammasome: molecular activation and regulation to therapeutics. *Nat. Rev. Immunol.* 19, 477-489. 10.1038/s41577-019-0165-0 31036962 PMC7807242

[DMM045732C40] Tang Y. and Le W. (2016). Differential Roles of M1 and M2 microglia in neurodegenerative diseases. *Mol. Neurobiol.* 53, 1181-1194. 10.1007/s12035-014-9070-5 25598354

[DMM045732C41] Tang L., Ma Y., Liu X.-L., Chen L. and Fan D.-S. (2019). Better survival in female SOD1-mutant patients with ALS: a study of SOD1-related natural history. *Transl. Neurodegener.* 8, 2 10.1186/s40035-018-0142-8 30637102 PMC6325854

[DMM045732C42] Walker D. G. and Lue L.-F. (2015). Immune phenotypes of microglia in human neurodegenerative disease: challenges to detecting microglial polarization in human brains. *Alzheimers Res. Ther.* 7, 56 10.1186/s13195-015-0139-9 26286145 PMC4543480

[DMM045732C43] West M. J., Slomianka L. and Gundersen H. J. G. (1991). Unbiased stereological estimation of the total number of neurons in the subdivisions of the rat hippocampus using the optical fractionator. *Anat. Rec.* 231, 482-497. 10.1002/ar.1092310411 1793176

[DMM045732C44] White M. A., Kim E., Duffy A., Adalbert R., Phillips B. U., Peters O. M., Stephenson J., Yang S., Massenzio F., Lin Z. et al. (2018). TDP-43 gains function due to perturbed autoregulation in a Tardbp knock-in mouse model of ALS-FTD. *Nat. Neurosci.* 21, 552-563. 10.1038/s41593-018-0113-5 29556029 PMC5884423

[DMM045732C45] Wooley C. M., Sher R. B., Kale A., Frankel W. N., Cox G. A. and Seburn K. L. (2005). Gait analysis detects early changes in transgenic SOD1(G93A) mice. *Muscle Nerve* 32, 43-50. 10.1002/mus.20228 15880561 PMC1350398

[DMM045732C46] Yiangou Y., Facer P., Durrenberger P., Chessell I. P., Naylor A., Bountra C., Banati R. R. and Anand P. (2006). COX-2, CB2 and P2X7-immunoreactivities are increased in activated microglial cells/macrophages of multiple sclerosis and amyotrophic lateral sclerosis spinal cord. *BMC Neurol.* 6, 12 10.1186/1471-2377-6-12 16512913 PMC1413551

