# Supplementary material for: Chronic administration of P2X7 receptor antagonist JNJ-47965567 delays disease onset and progression, and improves motor performance in ALS SOD1G93A female mice
Source: Dis Model Mech. 2020 Oct 30;13(10):dmm045732. doi: 10.1242/dmm.045732 (PMC7648608; doi:10.1242/dmm.045732)
Supplement: Supplementary information [file dmm-13-045732-s1.pdf]

SUPPLEMENTARY INFORMATION

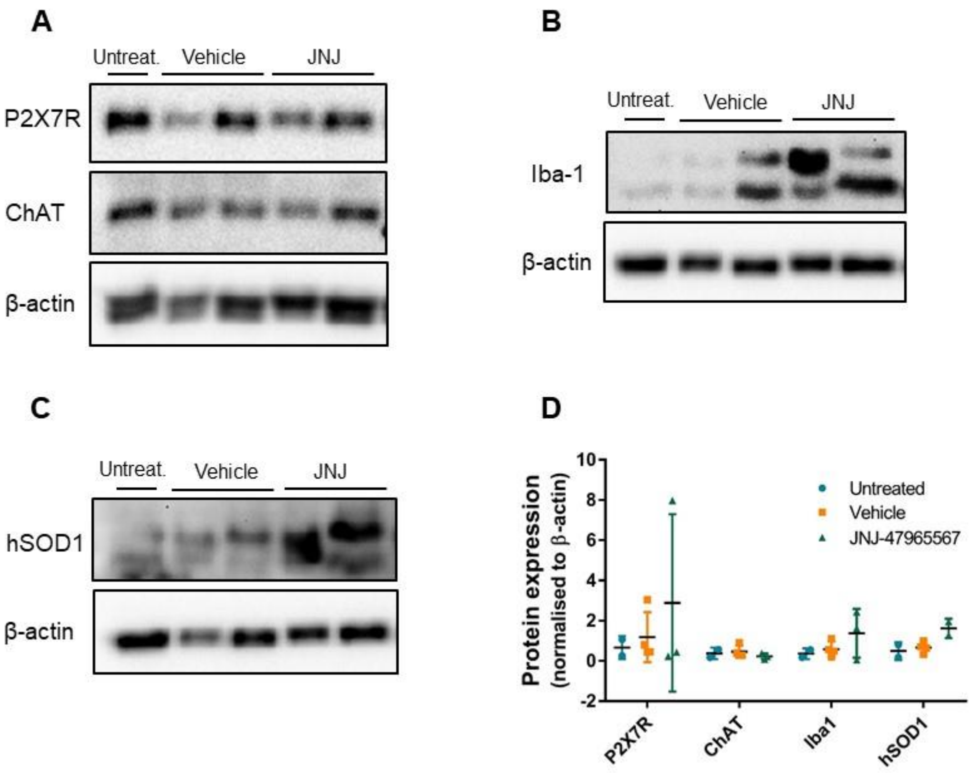

Fig S1. Protein expression analysis of spinal cord homogenates extracted from end-point females using Western Blot. Antibodies against P2X7R; choline acetyltransferase (ChAT), marker of motor neuron viability (**A**); Iba-1, marker of microglia (**B**); and mutated human SOD1 (**C**) were used. Results are presented in panel **D** as mean ± SD. Data were analyzed with one-way ANOVA followed by Tukey's multiple comparisons test. Differences were considered as statistically significant when $p<0.05$.

Disease Models & Mechanisms • Supplementary information
